# Coculture of Primary Motor Neurons and Schwann Cells as a Model for *In Vitro* Myelination

**DOI:** 10.1038/srep15122

**Published:** 2015-10-12

**Authors:** Sujin Hyung, Bo Yoon Lee, Jong-Chul Park, Jinseok Kim, Eun-Mi Hur, Jun-Kyo Francis Suh

**Affiliations:** 1 Center for Bionics, Korea Institute of Science and Technology (KIST), Seoul, Korea; 2 Brain Korea 21 PLUS Project for Medical Science, Yonsei University, Seoul, Korea; 3 Center for Neuroscience, Korea Institute of Science and Technology (KIST), Seoul, Korea; 4 Department of Neuroscience, Korea University of Science and Technology, Daejeon, Korea

## Abstract

A culture system that can recapitulate myelination *in vitro* will not only help us better understand the mechanism of myelination and demyelination, but also find out possible therapeutic interventions for treating demyelinating diseases. Here, we introduce a simple and reproducible myelination culture system using mouse motor neurons (MNs) and Schwann cells (SCs). Dissociated motor neurons are plated on a feeder layer of SCs, which interact with and wrap around the axons of MNs as they differentiate in culture. In our MN-SC coculture system, MNs survived over 3 weeks and extended long axons. Both viability and axon growth of MNs in the coculture were markedly enhanced as compared to those of MN monoculture. Co-labeling of myelin basic proteins (MBPs) and neuronal microtubules revealed that SC formed myelin sheaths by wrapping around the axons of MNs. Furthermore, using the coculture system we found that treatment of an antioxidant substance coenzyme Q10 (Co-Q10) markedly facilitated myelination.

Myelin provides an electrically insulating material that forms a multilayered sheath around an axon of a neuron. In the peripheral nervous system (PNS), myelin sheath is generated by Schwann cells (SCs), whereas in the central nervous system (CNS), myelin is produced by oligodendrocytes. The myelin wrapping of axons by SCs and oligodendrocytes not only facilitates the conduction of action potentials in the nervous system, but also protects axons and provides nutritional support to associated axons. As various defects in myelination, such as hypomyelination, delayed myelination, or demyelination, are known to cause serious disorders in the nervous system^1^,^2^,^3^,^4^, understanding how SCs and oligodendrocytes initially produce myelin during development and remyelinate axons in the diseased nervous system is of significant clinical interest. In many cases of nerve repair after physical injury, an incomplete myelination of regenerated nerve fibers causes malfunction of repaired nerve^5^.

To understand the process of myelination and remyelination, several *in vitro* models have been developed. Methods to culture SCs and oligodendrocytes have been established and extensively applied to investigate the biology of myelinating glia^6^,^7^,^8^, and these cultures greatly improved the understanding of how SCs and oligodendrocytes differentiate and mature. In addition to culture systems comprising pure SCs or oligodendrocytes, coculture systems composed of both neurons and myelinating glia should provide insights into the interaction between myelination glial cells and axons as well as the myelinating process. To this end, previous studies described coculture systems of oligodendrocytes and hippocampal neurons^8^, SCs and sensory neurons (from dorsal root ganglia)^6^, or oligodendrocytes and retinal ganglion cells^9^. Recently, Gingras *et al.*^10^ reported a method for culturing primary motor neurons (MNs) on collagen-chitosan sponges seeded with fibroblasts or with fibroblasts and Schwann cells, which enabled long-term culture (lasting more than a month) of MNs. However, the protocol required 21 days of culture before the addition of MNs, and efficient axon outgrowth could be achieved by the addition of high concentrations of several exogenous neurotrophic supplements, including BDNF, neurotrophin-3, glial-derived neurotrophic factor, and ciliary neurotrophic factor. On the other hand, Haastert *et al.*^11^ reported that when cultured on a feeder layer of SCs, MNs without supplementary support of neurotrophic factors matured in culture for 20 days *in vitro* (DIV), but that the fraction of MNs gradually reduced from higher than 70% on DIV 3 down to 12% by DIV 20. In addition, in the coculture model of MNs and SCs, MNs matured with enlarged somata but myelination of MN axons by SCs was not achieved during the culture period.

In the present study, we report a coculture system comprising primary mouse MNs and SCs. In our system, MNs survived for at least 3 weeks in culture and more importantly, SCs differentiated and formed myelin sheath around the axons of MNs. In the coculture, neurons exhibited higher viability and extended longer axons as compared to MN monoculture. By employing standard cell biological and biochemical approaches, we have confirmed that SCs myelinate MN axons within 3 weeks of coculture. The formation of myelin sheath was confirmed at the ultrastructural level. Furthermore, using the MN-SC coculture, we show that treatment with coenzyme Q10 facilitates myelination.

## Results

### Establishment of a MN-SC Coculture System

**Figure 1:**
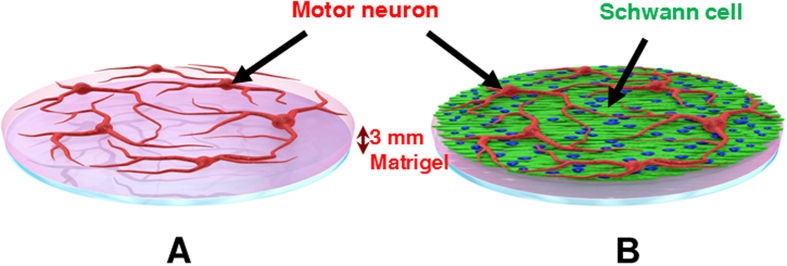
Schematic illustration of MN monoculture and MN-SC coculture on a matrigel. (**A**) MNs cultured on a coverslip coated with growth factor-reduced matrigel. (**B**) MNs cultured on a SC feeder layer that grew up to 90% confluency.

**Figure 2:**
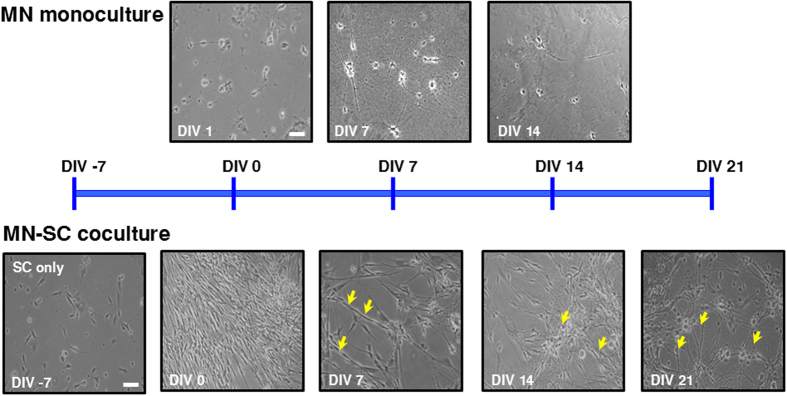
Flowchart of the differentiation of MNs in culture. Examples of light microscopic images of MN monoculture and MN-SC coculture at different stages are shown. In the MN monoculture, purified neurons form synapses at DIV 7, but viability greatly reduces by DIV 14. In the MN-SCs coculture, SCs are cultured for 7 days, and then MNs are grown on top of the SC feeder layer. MNs in the coculture survive for at least 3 weeks and motor axon diameter increases (arrows, yellow). All images are at the same scale. Scale bar, 50 µm.

**Figure 3:**
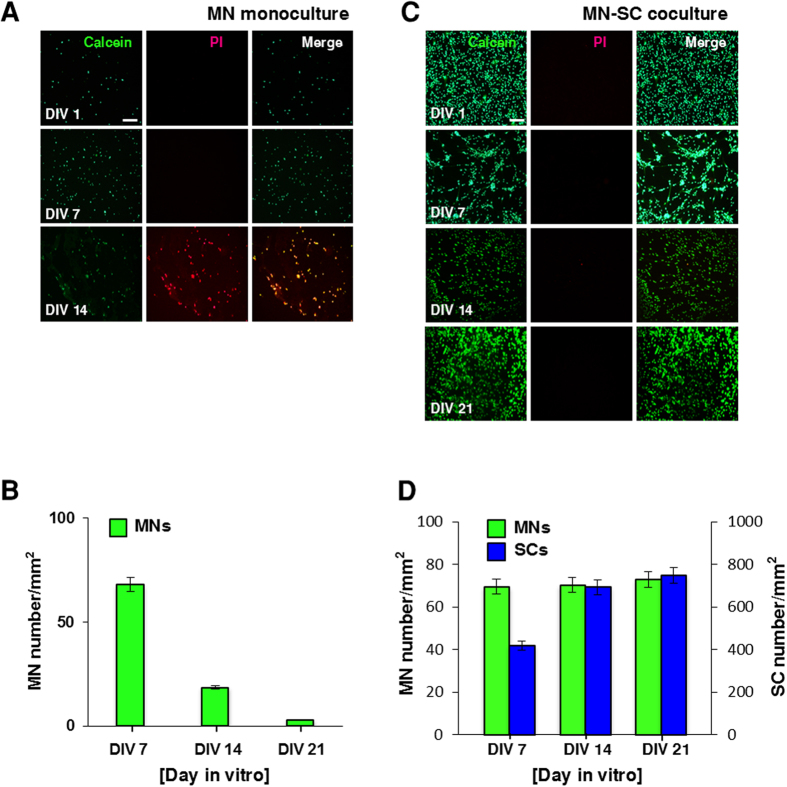
Cell viability in MN monoculture (**A** and **B**) and MN-SC coculture (**C** and **D**). Viability was assessed by double-staining with calcein-AM and propidium iodide (PI) at DIV 0, 7, 14, or 21, as indicated (**A** and **C**). Samples were then fixed and immunostained with DAPI and NeuN antibodies (**B** and **D**). Representative images (**A** and **C**) and quantification of NeuN-positive MNs are shown (**B** and **D**). (n = 5), Scale bar, 100 µm.

To develop a long-term culture model of MNs, SCs were harvested from the sciatic nerves of postnatal day 4 (P4) mice and cultured on approximately 3 mm-thick matrigel. SCs were cultured for a week until cells formed a confluent feeder layer. On top of SC feeder layer, purified MNs obtained from the ventral horns of gestation day 14 embryos (E14) were seeded and cocultured (Fig. 1B, MN-SC coculture). As a control, MNs were cultured directly on matrigel without prior culture of SCs (Fig. 1A, MN monoculture). In the MN monoculture, MNs extended axons up to DIV 7, but neurons gradually degenerated thereafter and by DIV 14, few MNs survived (Fig. 2). By contrast, in the MN-SC coculture, neurons differentiated and developed neurites and remained intact up to at least DIV 21. On DIV 3, we were able to detect migration of SCs toward the axons of MNs and on DIV 7, we observed nuclei of SCs around the axons. We also compared cell viability in MN monoculture and MN-SC coculture systems by using Live-Dead cell staining kit and NeuN-DAPI double antibody staining methods (Details in Methods section). In the MN monoculture, vast majority of neurons (96%) failed to survive (Fig. 3A,B). The MN-SC coculture, however, showed almost no cell death up to DIV 21 (Fig. 3C). Furthermore, the viable MN cell number was maintained throughout the culture, whereas the viable SC cell number steadily increased up to DIV 21 (Fig. 3D), suggesting that SCs play a critical role in supporting the survival of MNs.

**Figure 4:**
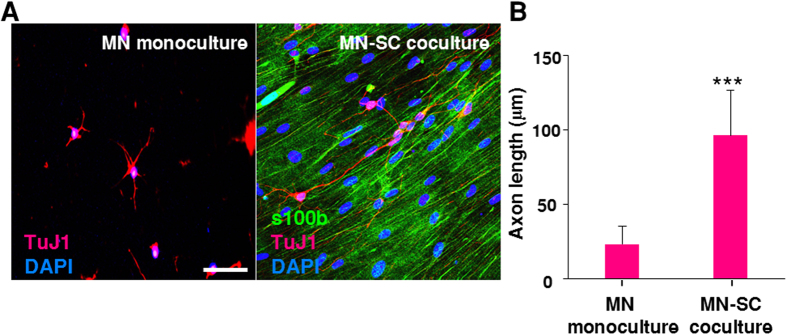
Axonal outgrowth in MN monoculture and MN-SC coculture. (**A**) Confocal images of MN monoculture and MN-SC coculture are shown. Cells were fixed and stained for s100β, TuJ1 and nucleus (DAPI), as indicated, at DIV 2, and lengths of axons were measured. Scale bar, 50 µm. (**B**) Each bar represents mean ± S.E. value. (n= 100) *** p < 0.001.

We next quantitatively examined the effects of SCs on axon outgrowth of MNs using TuJ1 staining for the nerve fibers and ImageJ to quantify the fiber length (Fig. 4). When axon length was measured at 2 days after plating MNs (Fig. 4A), average axon length of MNs grown in the MN-SC coculture system was about 4 times longer than that of MN monoculture. In the MN-SC coculture, axons grew at a rate of about 50 μm per day, which is consistent with a previous report^7^. Taken together, these results suggest that SCs are essential for the survival and neurite outgrowth of MNs.

### Myelination of Motor Neurons

**Figure 5:**
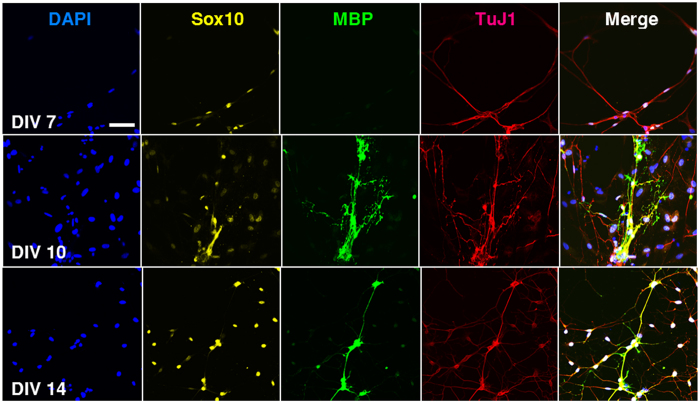
Differentiation of neurons and SCs in MN-SC coculture. Confocal images of MN-SC coculture are shown. Cells were fixed and stained for Sox10, MBP, TuJ1 and nucleus (DAPI) at DIV 7, 10, and 14, as indicated. Note the expression of MBP protein around axons at DIV 10 and 14. Scale bar, 50 µm.

**Figure 6:**
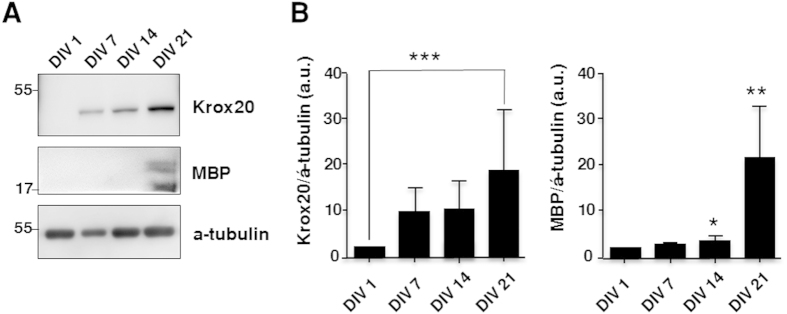
Increased expression of Krox20 and MBP protein in the MN-SCs coculture. The levels of Krox20 and MBP were determined by western blot analysis at DIV 1, 7, 14, and 21, as indicated. Representative blots (**A**) and quantification (**B**) of Krox20 and MBP levels are shown. Protein levels were normalized against the level of α-tubulin, which was used as a loading control. Each bar represents the average expression level of a protein of interest normalized to that of DIV 1 (n=5).

**Figure 7:**
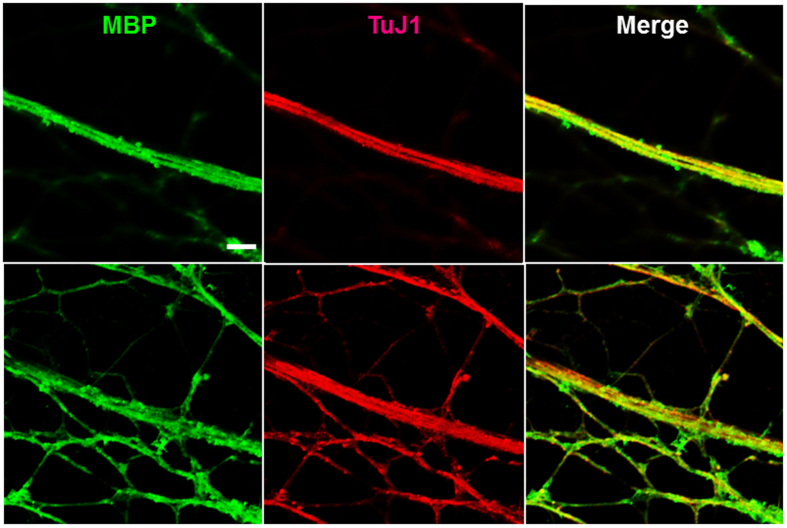
Formation of compact myelin sheaths in the MN-SCs coculture model. At DIV 21, cultured cells were observed by confocal microscopy after immunolabeling with anti-MBP (1:500, green) and anti-TuJ1 antibodies (1:1000, red). Images shown at the top and bottom panels are serial images collected throughout z-sections. Scale bar, 5 μm.

**Figure 8:**
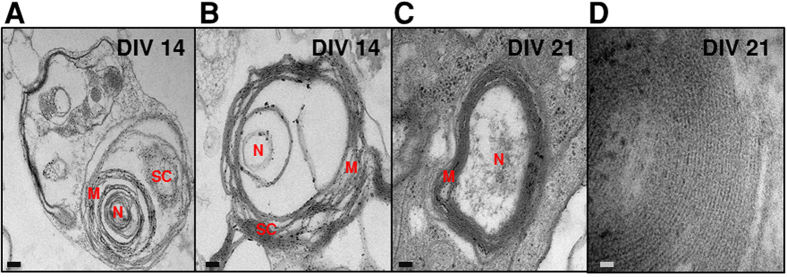
Myelination of axons in the MN-SC coculture model. The extent of myelin sheaths was analyzed by transmission electron microscopy at DIV 14 and 21. Micrographs from DIV 14 show SCs loosely wrapping around the nerve fiber (**A, B**), whereas those from DIV 21 clearly show the formation of compact myelin sheaths (**C**). Representative image presented in D shows a heavily myelinated nerve fiber. Scale bar, 200 nm (**A-C**), 20 nm (**D**). N, nerve fiber; m, myelin sheaths.

To investigate if SCs differentiate and more importantly myelinate MN axons in our MN-SC coculture system, cultures were examined for expression of markers of SC differentiation and myelination, such as myelin basic protein (MBP), SRY-related HMGbox-10 (Sox10) and Krox20. We found that the expression of Sox10, a transcription factor which controls myelination in the PNS^12^, was detected in the soma of SCs on DIV 7 and increased thereafter (Fig. 5). Expression of MBP was detected on DIV 10, and eventually became highly localized along the axon fiber by DIV 14 (Fig. 5, green). Western blot analysis confirmed the expression of MBP and Krox20, a transcription factor induced by the activation of Sox10. Krox20 is known to suppress the immature state and promote myelination^13^,^14^. We observed an increase of both MBP and Krox20 protein levels over time (Fig. 6), consistent with our immunocytochemistry results (Fig. 5). Next, we examined myelin sheath formation on DIV 21 in detail by confocal microscopy and transmission electron microscopy (TEM). Z-stack images of confocal microscopy confirmed tight interaction between MBP and axons of MNs (Fig. 7, Supplemental movie 1). By taking TEM images, we confirmed that SCs formed loose sheaths by DIV 14, which could be the premyelination stage (Fig. 8A,B), and that SCs tightly wrapped the nerve fiber by DIV21, which could be the completed myelination stage (Fig. 8C,D). The thickness of compact myelin sheaths was approximately 0.2 μm at DIV 21.

### Distinct Stages of Myelination Observed in the MN-SC Coculture System

**Figure 9:**
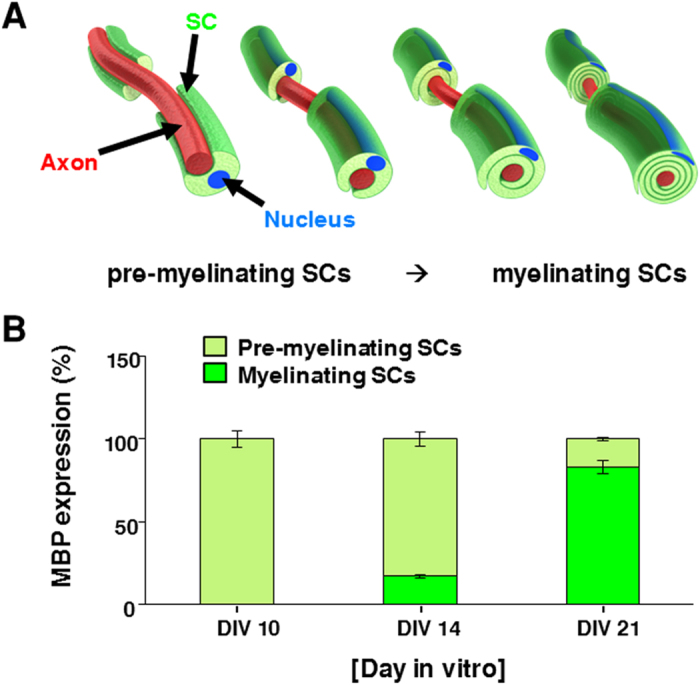
Pre-myelinating and myelinating SCs. (**A**) The process of myelination. SCs envelop an axon and continue to produce myelin. SCs then wrap their plasma membrane loosely around an axon, yielding in multiple successive layers. Membrane wrappings are compacted into a tightly packed insulation coat. (**B**) SCs in culture were fixed and stained for MBP at DIV 10, 14, and 21, as indicated. MBP-expressing SCs were categorized into two groups, pre-myelinating and myelinating (n=5).

It is well-known that oligodendrocytes in the CNS differentiate from pre-myelinating to myelinating stage and the transition is controlled by several intrinsic and extrinsic signals^15^. Likewise, in our MN-SC coculture system, SC myelination proceeded through two distinct stages, which could be distinguished on the basis of MBP expression pattern and interaction with axons as mentioned above in the TEM images. In the pre-myelinating stage, expression of MBP in SCs was relatively widespread, whereas in the myelinating stage, MBP expression in SCs was tightly localized along the axons. By counting the number of these two different types of SCs under confocal microscope, we found that while most SCs were at pre-myelinating stage when observed on DIV 10, the number of myelinating SCs gradually increased in culture and reached 80% by DIV 21 (Fig. 9B).

### Promotion of MN myelination by Coenzyme Q10

**Figure 10:**
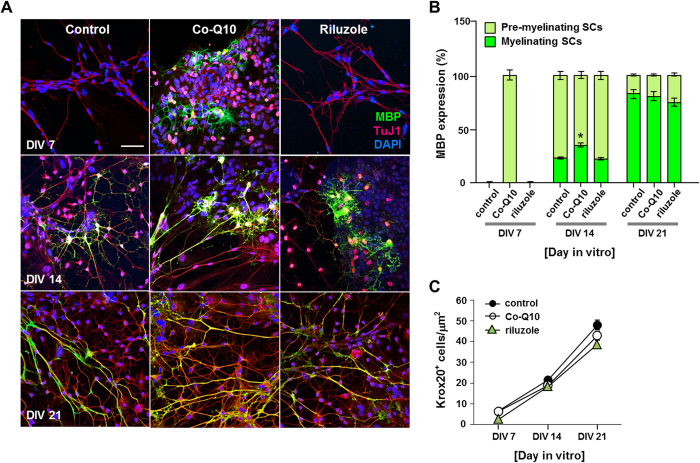
Effects of drug treatment on myelination. Comparison of MBP expression in MN-SC coculture treated with coenzyme Q10 (Co-Q10) or riluzole using immunocytochemistry. Note the marked elevation of pre-myelination in cultures treated with Co-Q10 at DIV 7. (n=5), * p < 0.05, Scale bar, 20 μm.

We next treated our coculture samples with drugs implicated in the treatment of neurological diseases. Samples were treated with riluzole (2-amino-6-(trifluoromethoxy) benzothiazole), an FDA-approved drug for the treatment of amyotrophic lateral sclerosis (ALS)^16^, a debilitating neuromuscular disease which involves the death of motor neurons, or with coenzyme Q10 (Co-Q10) (1,4-benzoquinone, also known as ubiquione), an antioxidant and mitochondrial cofactor, which has been considered as a potential therapeutic agent for demyelinating and neurodegenerative diseases^17^,^18^. As shown in Fig. 10A,B, Co-Q10 treatment facilitated MBP expression and increased the number of myelin-forming SCs as compared to control. When observed on DIV 7, MBP expression was detected only in Co-Q10-treated cultures but not in control or riluzole-treated cultures. By DIV 14, MBP expression was also detected in control and riluzole-treated cultures, but the percentage of myelinating SCs was lower as compared to Co-Q10-treated cultures. By DIV 21, all cultures expressed MBP. In all cultures, we detected an increase of Krox20 expression, demonstrating that SCs differentiate properly in our MN-SC coculture system and that treatment of riluzole or Co-Q10 did not affect the transition from pre-myelinating to myelinating stage *per se* but perhaps control later stages of SC myelination (Fig. 10C).

## Discussion

Several *in vitro* models have been previously developed to study the cellular and molecular mechanisms involved in the myelination process. Sensory neurons from dorsal root ganglia^6^,^7^,^19^,^20^ or retinal ganglia^21^ were cocultured with SCs to induce the formation of myelin sheath *in vitro.* Several *in vitro* models for MN culture have also been introduced^10^,^11^,^22^,^23^,^24^, and the findings from these studies have confirmed that addition of exogenous neurotrophic supplements and/or a coculture with SCs or fibroblasts was critical for the survival and neurite outgrowth of MNs cultured *in vitro*. MN cultures treated with astrocyte conditioned medium^23^ or insulin-like growth factor-1^22^ could specifically enhance the extent and rate of axonal outgrowth from murine corticospinal MNs. However, to the best of our knowledge, no culture method to date has recapitulated the myelination process of MN by SCs *in vitro*. Moreover, previous studies have attempted to culture MNs by using NycoPrep^TM^, OptiPrep^TM^, or immunopanning dishes, but the purified MNs frequently failed to survive long-term^11^,^24^,^25^. In this study, we have aimed at constructing a MN-SC coculture system to model both the axon outgrowth and the myelination process of MN.

Here we have confirmed that SCs play an essential role in increasing the viability and promoting axonal outgrowth of MNs. Consistent with our results, several groups have reported that SCs enhance long-term viability of peripheral neurons and protect the axonal integrity. Meyer zu Horste *et al.*^26^ have reported that dysfunction of SCs leads to peripheral neuropathies, and Viader *et al.*^27^ have found that mitochondrial function in SCs is essential not only for neuronal survival but also for normal function of peripheral nerves. Similarly, glial cells have been shown to play a vital role for the growth and stability of developing synaptic structure as well as for proper functioning of neuromuscular junction^28^. With regard to nerve injury, it is well documented that SCs play a role in promoting recovery of the injured PNS by providing beneficial environment to the injured neurons^29^,^30^. SCs de-differentiate, proliferate, and form a Bands of Büngner at the injury site, by which they guide the regrowth of the proximal axons towards the target^31^. Our results, along with the previous reports, support the notion that SCs play an essential role in controlling the viability, axon growth, and perhaps the functions of MNs.

Our culture enables monitoring of the pre-myelinating and myelinating stages of SCs *in vitro*. Signals released from Schwann cells are critical for myelination of peripheral nerve fibers^12^. NRG1 (neuregulin) in the axonal membrane is essential for initiating the myelination process by binding to ErbB (erythroblastic leukemia viral oncogene homolog) family receptors expressed in SCs, and SC-derived signals are thought to primarily control the wrapping process of myelin around the axon. Among them, Krox20/Egr2 (early growth response-2), which functions downstream of Sox10 (SRY-related HMGbox-10) and is activated by Oct6 (octamer-binding transcription factor-6), is essential for activating a number of myelin genes and maintaining the myelinated state by blocking inhibitors of myelination. Our results in Fig. 5 show that Sox10 expression at DIV 7 preceded MBP expression, which was observed at DIV 10. The results in Fig. 6 also show a gradual increase of Krox20 first detected on DIV 7, followed by a strong expression at a later stage (DIV 21). At later stages, MBP was highly localized along the axon fibers (Fig. 7). Our findings are consistent with the well-documented hierarchy of SC transcription factors controlling myelination (reviewed by Pereira *et al.*^12^). In our culture, we were able to confirm the sequential process of myelination, which was initiated by migration of SCs toward axons, followed by further differentiation of SCs, as evidenced by increased expression of MBP. Finally, SCs wrapped around axons, leading to the formation of thick myelin sheaths.

In the present study, we examined the efficacy of two candidate drugs, which we thought, might promote myelination of nerve fibers. We tested the effect of riluzole, a drug which is thought to protect neurons by decreasing the level of glutamate, and approved by FDA and clinically used for the treatment of ALS^32^. We also tested the effect of Co-Q10, a vitamin-like antioxidant substance that enhances ATP production in mitochondria. We found that while riluzole had no significant effect on myelination of MN axon fiber, Co-Q10 facilitated MBP expression and enhanced myelination. No positive effect of riluzole confirmed in our study could be explained within the context of the controversy about the efficacy of riluzole treatment of ALS^33^. SCs treated with Co-Q10 expressed MBP at an earlier stage (DIV 7), implying that Co-Q10 might accelerate myelination of nerve fibers. MNs seem to have higher energy demand and metabolic rate due to their increased mitochondrial activity in comparison to other neurons^34^. The stimulating effect of Co-Q10 found in our study could be due to its ATP synthesizing ability as electron carriers^35^ or the antioxidant nature of Co-Q10^36^,^37^, or a combination of both. The exact mechanism and efficacy of Co-Q10 warrants further studies.

## Conclusions

In conclusion, we report a simple and reproducible MN-SC coculture system derived from mouse neurons and glia. In the coculture, SCs enhanced cell viability and promoted axonal outgrowth of MNs. Moreover, SCs differentiated and transformed from pre-myelinating to myelinating stage as evidenced by the gradual increase in MBP expression and the localization of MBP signals along axon fibers. Furthermore, using the MN-SC coculture, we show that Co-Q10 accelerates myelination.

## Methods

### Cell Preparation

#### Schwann Cell (SC) Culture

All animal experiments have been conducted in accordance with protocols approved by the Institutional Animal Care and Use Committee (IACUC) at the Korea Institute of Science and Technology. SCs were isolated from sciatic nerves of CD-1 mice (P4), as previously described^6^. Explants were incubated in Ca^2+^- and Mg^2+^-free phosphate-buffered saline (PBS, Lonza) containing 0.05% trypsin (Sigma) and 0.05% collagenase A (Roche) for 30 min at 37 °C, followed by centrifugation at 190 g for 5 min. The pellets containing tissue fragments and cells were then washed three times with high glucose—DMEM (Gibco) containing 10% horse serum (HS) (Gibco). The harvested SC pellets were suspended in SC basic culture medium composed of high glucose DMEM, 10% HS, 4 mM L-glutamine (L-gln, Invitrogen), 100 units/ml penicillin/streptomycin (P/S, Sigma), 2 ng/ml human heregulin beta-1(Sigma), and 0.5 μM forskolin (Sigma). After suspended, SCs were seeded on 12 mmϕ coverslips at a density of 3 × 10^4^ cells per coverslip. Prior to seeding SCs, the coverslips were coated with growth factor-reduced matrigel (3 mm thickness on average). On DIV 4, complement-mediated cytolysis was performed to remove fibroblasts from the SC culture. Briefly, the cells were lightly washed with 20 mM HEPES (T&I) in Ca^2 + ^- and Mg^2 + ^-free HBSS solution (Invitrogen), followed by washing with HMEM (high-glucose DMEM containing 20 mM HEPES buffer, 10% HS, 4 mM L-gln, 100 units/ml P/S). After washing, 4 ng/ml anti-mouse CD90 (Serotec) in HMEM was added for 15 min at 37 °C, and 200 μl of rabbit human leucocyte-associated antigens A, B, C (HLA-ABC) complement sera (Millipore) was added for 2 hrs at 37 °C. Cytolysis was terminated by washing cells with 20 mM HEPES buffer in HBSS. After the cytolysis, SCs were cultured for another three days in the SC growth medium containing SC basic culture medium (described above) supplemented with 10 ng/ml of bFGF (Sigma) and 20 μg/ml of bovine pituitary extract (Sigma) to promote cell proliferation.

#### Motor Neuron (MN) Culture

MNs were harvested from the spinal cord of CD-1 mice (E14 fetus) as previously described^24^ with some modifications. Ventral horns of the spinal cord (L1-L6 segment) were each collected in HBSS and 1% TLR trypsin (Worthington) was added for 15 min in a 37 °C water bath before treating with 1% TLR trypsin inhibitor (Sigma). To extract a purified population of MNs, cells dissociated from the explant were treated for 45 min at room temperature with a selective immuno-panning dish prepared with p75^NTR^ antibody solution (1:5000, Abcam) in 10 mM Tris-HCl (pH 9.5, T&I). Suspensions, including nerve fragments and p75^NTR^-negative cells, were removed from the panning dish by washing three times with pre-warmed neurobasal medium containing Glutamax 1 (Gibco). MNs bound to the panning dish were then rinsed with depolarization solution containing 0.8% sodium chloride, 30 mM potassium chloride, and 2 mM calcium chloride (Merk) in distilled water for 10 sec, and gently collected into the MN culture medium composed of neurobasal medium (Invitrogen), 10% HS, Glutamax 1, B27 supplement (Gibco), 1 μM β-mercaptoethanol (Sigma), and 10 ng/ml BDNF (Gibco). MNs were collected by gentle pipetting, and were then centrifuged for 5 min at 400 g. After centrifugation, the pellets were re-suspended in the MN culture medium.

#### Coculture of MNs and SCs

MNs were plated (1 × 10^4^ cells/coverslip) on top of a monolayer of SC culture which was prepared above (Fig. 1B). We performed that MN were seeded on SC feeder layer on which SCs cultured on growth factor reduced-matrigel to precede both migration and wrapping process of SCs around axons. In order to induce myelination, MN-SCs were first cultured in a coculture growth medium (neurobasal medium containing 2 ~ 10% HS, B27 supplement, 0.5 mM L-gln, 0.5 ~ 1 mM β-mercaptoethanol, 0.5 μM forskolin, 1 mg/ml bovine pituitary extract, 10 ng/ml BDNF) for 7 days, after which 25 ~ 50 μg/ml of ascorbic acid (Sigma) was supplemented for the subsequent culture. For MN monoculture, MNs were cultured directly on matrigel-coated coverslips without SC feeder layer (Fig. 1A). For drug treatment, cultures were treated with coenzyme Q10 (Co-Q10, 1 μM) or riluzole (0.1 μM), as indicated. At 24 hr after treatment, samples were fixed and subjected to immunostaining with the indicated antibodies. All culture medium was replenished three times a week.

### Measurement of Cell Viability

Live-Dead cell staining kit was used according to the manufacturer’s instructions (Abcam). Briefly, after 0, 7, and 14 days of monoculture or 7, 14, and 21 days of coculture, samples were labeled with mixture of solution A (Live-Dye) and solution B (PI) in the staining buffer for 15 min at 37 °C. The samples were then washed once with the staining buffer and examined under Olympus IX71 with U-RFL-T (detects FITC and TRITC). For NeuN immunostaining, cells were fixed with 4% paraformaldehyde (PFA) and treated with 0.2% Triton X-100 (Sigma), followed by blocking in 4% BSA (Millipore) at 4 °C overnight. Cells were then labeled with NeuN antibody (1:500, Abcam) for 24 hr at 4 °C, and were stained with Alexa Fluor® 488 donkey anti-mouse IgG (1:500, Invitrogen) for 1 hr at room temperature, followed by DAPI (Life Technologies) staining. The number of stained cells was counted from five random fields (Live-Dead cell, NeuN staining) of view under the microscope and statistical analysis on the cell viability was performed.

### Measurement of Axon Length

Cultures were fixed and labeled for TuJ1 to visualize axons of MNs, and the lengths of axons (n = 100) were measured by using ImageJ software.

### Immunocytochemistry

After 7, 14, and 21 days of coculture, all samples were fixed with 4% paraformaldehyde (PFA) for 15 min at room temperature and washed twice with PBS. The fixed samples were then soaked in PBS containing 0.2% Triton X-100 (Sigma), followed by 4% BSA (Millipore) at 4°C overnight and labeled with primary antibodies, which included anti-Sox10 (1:500, Abcam), anti-s100β (1:500, Abcam), anti-β-III Tubulin (Tuj1, 1:1000, Abcam), and anti-myelin basic protein (MBP, 1:500, Abcam). All samples with primary antibodies were incubated in 1% BSA with PBS at 4°C overnight. The samples were then washed with PBS containing 1% BSA twice, and incubated with goat anti-chicken IgY H&L (1:1000, Abcam), goat anti-rabbit IgG H&L (1:500, Abcam), or Alexa Fluor® 488 donkey anti-mouse IgG (1:500, Invitrogen). The nuclei of cells were stained with DAPI (Life Technologies) for 15 min at room temperature. Fluorescence of the culture was visualized under a confocal microscope (Zeiss LSM 700).

### Western Blot Assays

Expressions of MBP and Krox20 were quantitatively analyzed by using western blot analysis. The cells at DIV 1, 7, 14, and 21 were lysed in RIPA buffer containing 1% SDS and protease inhibitor (aprotinin, leupeptin, pepstatin A, PMSF). The protein concentration of cell lysates was measured by using Bradford assay. The samples were prepared in an SDS sample buffer, heated for 3 min at 98 °C, and 6 μg of the protein from each sample were loaded in SDS loading buffer and then transferred to a PVDF membrane. The membranes were blocked in 2.5% skim milk for 1 hr, followed by incubation with anti- rabbit Krox20 (1:1000, Abcam) or anti- rat MBP (1:500, Abcam) antibodies at 4 °C overnight. The membranes were washed three times in TBST and incubated with goat anti-rabbit and anti- rat IgG conjugated to horseradish peroxidase (1:1000, Sigma) for 2 hrs. Bands were visualized by using ECL system. The intensity of the blots was quantified with ImageJ software.

### Transmission Electron Microscopy

At DIV 14, 21, cultures were fixed with 4% glutaraldehyde in PBS and stored at 4 °C overnight, and postfixed with 1% osmium tetroxide for 30 min. Samples were embedded in pre epoxy resin before dehydration with a series of ethanol solutions (70%, 80%, 85%, 90%, 95%, 100%). Sections were taken between 70 ~ 80 nm using Ultramicotome (Leica, Ultra Cut C) and picked up on Cu grids, stained in uranyl acetate and lead citrate. Images were acquired with the Cryo-TEM (FEI, CryoTecnai F20).

### Quantitative Analysis of Pre-myelinating and Myelinating Schwann Cells

During coculture, cultures immunostained with anti-MBP antibodies showed two distinct patterns of MBP expression: pre-myelinating SCs showed relatively wide-spread expression of MBP adjacent to the axons while myelinating SCs showed highly localized expression of MBP along the axons. Cocultures were stained with MBP and TuJ1 at DIV 10, 14, and 21 and the proportion of these two types was calculated from six different fields of view under confocal microscope.

### Statistical Analysis

Average data are shown as mean ± standard error and the comparison between different groups were performed by using repeated measurement analysis of variance (ANOVA), and statistical significance was set at a value of *P < 0.05, ***p < 0.001.

## Additional Information

**How to cite this article**: Hyung, S. *et al.* Coculture of Primary Motor Neurons and Schwann Cells as a Model for *In Vitro* Myelination. *Sci. Rep.* **5**, 15122; doi: 10.1038/srep15122 (2015).

## Supplementary Material

Supplementary Information

Supplementary Movie
