# Supplementary material for: Coculture of Primary Motor Neurons and Schwann Cells as a Model for In Vitro Myelination
Source: Sci Rep. 2015 Oct 12;5:15122. doi: 10.1038/srep15122 (PMC4601011; doi:10.1038/srep15122)
Supplement: Supplementary Information [file srep15122-s1.pdf]

Coculture of Primary Motor Neurons and Schwann Cells as a Model for *In Vitro* Myelination

Sujin Hyung[a,b], Bo Yoon Lee[c,d], Jong-Chul Park[b], Jinseok Kim[a], Eun-Mi Hur[c,d], and Jun-Kyo Francis Suh[a]

[a] Center for Bionics, Korea Institute of Science and Technology (KIST), Seoul, Korea
[b] Brain Korea 21 PLUS Project for Medical Science, Yonsei University, Seoul, Korea
[c] Center for Neuroscience, Korea Institute of Science and Technology (KIST), Seoul, Korea
[d] Department of Neuroscience, Korea University of Science and Technology, Daejeon, Korea

Figure Captions

Figure 1. Schematic illustration of MN monoculture and MN-SC coculture on a matrigel. (A) MNs cultured on a coverslip coated with growth factor-reduced matrigel. (B) MNs cultured on a SC feeder layer that grew up to 90% confluency.

Figure 2. Flowchart of the differentiation of MNs in culture. Examples of light microscopic images of MN monoculture and MN-SC coculture at different stages are shown. In the MN monoculture, purified neurons form synapses at DIV 7, but viability greatly reduces by DIV 14. In the MN-SCs coculture, SCs are cultured for 7 days, and then MNs are grown on top of the SC feeder layer. MNs in the coculture survive for at least 3 weeks and motor axon diameter increases (arrows, yellow). All images are at the same scale. Scale bar, 50 μm.

Figure 3. Cell viability in MN monoculture (A and B) and MN-SC coculture (C and D). Viability was assessed by double-staining with calcein-AM and propidium iodide (PI) at DIV 0, 7, 14, or 21, as indicated (A and C). Samples were then fixed and immunostained with DAPI and NeuN antibodies (B and D). Representative images (A and C) and quantification of NeuN-positive MNs are shown (B and D). (n= 5), Scale bar, 100 μm.

Figure 4. Axonal outgrowth in MN monoculture and MN-SC coculture. (A) Confocal images of MN monoculture and MN-SC coculture are shown. Cells were fixed and stained for s100β, TuJ1 and nucleus (DAPI), as indicated, at DIV 2, and lengths of axons were measured. Scale

bar, 50 μm. (B) Each bar represents mean ± S.E. value. (n= 100) *** $p < 0.001$.

Figure 5. Differentiation of neurons and SCs in MN-SC coculture. Confocal images of MN-SC coculture are shown. Cells were fixed and stained for Sox10, MBP, TuJ1 and nucleus (DAPI) at DIV 7, 10, and 14, as indicated. Note the expression of MBP protein around axons at DIV 10 and 14. Scale bar, 50 μm.

Figure 6. Increased expression of Krox20 and MBP protein in the MN-SCs coculture. The levels of Krox20 and MBP were determined by western blot analysis at DIV 1, 7, 14, and 21, as indicated. Representative blots (A) and quantification (B) of Krox20 and MBP levels are shown. Protein levels were normalized against the level of α-tubulin, which was used as a loading control. Each bar represents the average expression level of a protein of interest normalized to that of DIV 1 (n=5).

Figure 7. Formation of compact myelin sheaths in the MN-SCs coculture model. At DIV 21, cultured cells were observed by confocal microscopy after immunolabeling with anti-MBP (1:500, green) and anti-TuJ1 antibodies (1:1000, red). Images shown at the top and bottom panels are serial images collected throughout z-sections. Scale bar, 5 μm.

Figure 8. Myelination of axons in the MN-SC coculture model. The extent of myelin sheaths was analyzed by transmission electron microscopy at DIV 14 and 21. Micrographs from DIV 14 show SCs loosely wrapping around the nerve fiber (A, B), whereas those from DIV 21 clearly show the formation of compact myelin sheaths (C). Representative image presented in

D shows a heavily myelinated nerve fiber. Scale bar, 200 nm (A-C), 20 nm (D). N, nerve fiber; m, myelin sheaths.

Figure 9. Pre-myelinating and myelinating SCs. (A) The process of myelination. SCs envelop an axon and continue to produce myelin. SCs then wrap their plasma membrane loosely around an axon, yielding in multiple successive layers. Membrane wrappings are compacted into a tightly packed insulation coat. (B) SCs in culture were fixed and stained for MBP at DIV 10, 14, and 21, as indicated. MBP-expressing SCs were categorized into two groups, pre-myelinating and myelinating (n=5).

Figure 10. Effects of drug treatment on myelination. Comparison of MBP expression in MN-SC coculture treated with coenzyme Q10 (Co-Q10) or riluzole using immunocytochemistry. Note the marked elevation of pre-myelination in cultures treated with Co-Q10 at DIV 7. (n=5), * $p < 0.05$, Scale bar, 20 μm.

Appendix A. Supplementary Movie

Supplementary Movie. Formation of compact myelin sheaths in the MN-SCs coculture. This movie shows detailed morphology of myelin sheath on DIV 21. Note that MBP expression is detected along axon fibers. Scale bar, 5 μm.

Supplementary Figure

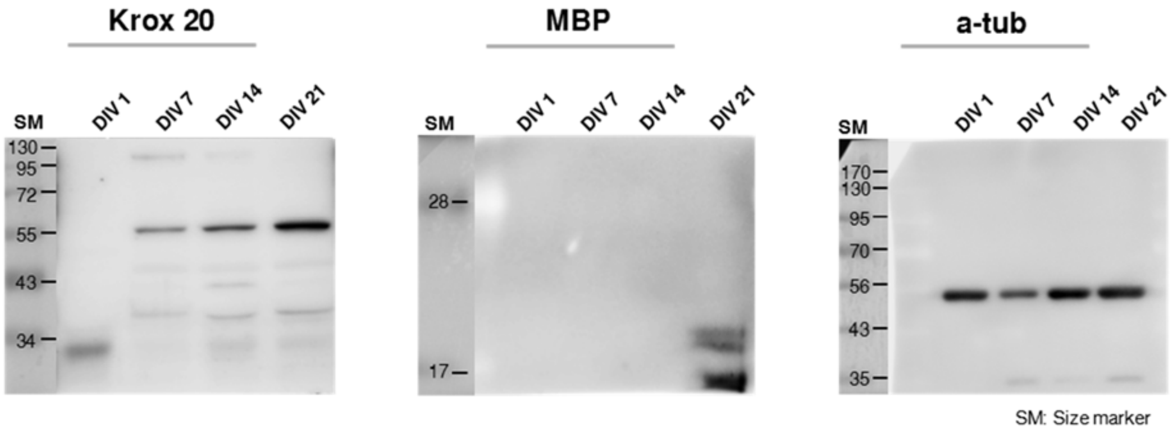

Figure S1. The full length blot image of Figure 6 in the main text. Representative blots of Krox20 and MBP levels are shown. The levels of Krox20 and MBP were determined by western blot analysis at DIV 1, 7, 14, and 21, as indicated.
